# Are costs optimized as scale-up of Choose to Move–an effective health-promoting intervention for older adults–proceeds?

**DOI:** 10.1186/s12966-025-01826-4

**Published:** 2025-11-13

**Authors:** Zoe Szewczyk, Heather M. Macdonald, Marina B. Pinheiro, Lindsay Nettlefold, Joanie Sims Gould, Heather A. McKay

**Affiliations:** 1 School of Public Health, Faculty of Medicine and Health, University of Sydney, Sydney, NSW Australia; 2 Active Aging Research Team, University of British Columbia, Vancouver, Canada; 3 Department of Family Practice, University of British Columbia, Vancouver, Canada; 4 Institute for Musculoskeletal Health, Sydney Local Health District, Gadigal Country, Sydney, Australia

**Keywords:** Cost, Cost analysis, Cost consequence, Healthy aging, Implementation, Scale-up

## Abstract

**Background:**

Few studies have examined costs of implementing evidence-based interventions (EBIs) as scale-up proceeds. Across four phases, we co-adapted and scaled up an effective EBI designed to promote older adults’ health (Choose to Move; CTM). Following formative evaluation (2015), Phases 1–2 (2016-17) comprised the CTM pilot and early scale-up. For Phase 3 (2018-20), we adapted CTM to establish “best fit” and support broad scale-up. In response to COVID-19 (2020), we adapted CTM for virtual delivery. For Phase 4 (2020-22), we adapted CTM to reduce resource use. We aimed to (1) identify, measure, and value costs of implementing CTM across four phases (7 years) of scale-up; and (2) analyze change in implementation costs alongside changes in intervention effect sizes to assess cost-consequence trends from Phases 1–2 through Phase 4.

**Methods:**

We conducted a trial-based cost and cost-consequence analysis of CTM Phases 1–2 through Phase 4 from a program provider perspective. Program costs were identified, measured, and valued using micro-costing techniques; variation in program cost was explored using scenario analyses. We compared Phase 4 intervention effects against those of Phases 1–2 and Phase 3 to examine how changes in implementation costs corresponded with changes in effect size.

**Results:**

For Phases 1–2, total cost ($CDN, 2024) of CTM implementation was $863,559 for 55 programs (534 participants; $1,617/participant). Phase 3 costs were $1,564,446 for 165 programs (1668 participants; $938/participant). Phase 4 costs were $760,983 for 135 programs (1278 participants; $595/participant), a reduction of 63% and 37% compared with Phases 1–2 and Phase 3, respectively. Compared with Phases 1–2, Phase 4 had a greater positive effect on social isolation but effect sizes for physical activity, mobility and loneliness were reduced. Phase 4 had a greater positive effect on physical activity and mobility in all participants, and loneliness among those < 75 years, compared with Phase 3.

**Conclusions:**

Costs associated with broad scale-up of EBIs are rarely investigated. We sought innovative ways to maximize impact of a health-promoting EBI, while minimizing costs. Our analysis highlights how strategic adaptations can enhance cost efficiency while improving intervention outcomes; this represents an emergent application of economic analysis within scale-up science.

**Trial registration:**

Retrospectively registered at ClinicalTrials.gov, NCT05678985 (CTM Phase 4) and NCT05497648 (CTM Phase 3).

**Supplementary Information:**

The online version contains supplementary material available at 10.1186/s12966-025-01826-4.

## Background

Optimization is the act of making the best or most effective use of a situation or resource. In public health, optimization is defined as “a deliberate, iterative, and data-driven process to improve a health intervention and/or its implementation to meet stakeholder-defined public health impacts within resource constraints” [1]. The Dynamic Sustainability Framework [2] suggests that “interventions should not be considered optimized until they have been implemented, tested, and refined in settings where they will be ultimately delivered”. To improve population health, it is important to design evidence-based interventions (EBIs) that are scalable, monitor scale-up as it progresses, and seek ways to optimize implementation as scale-up proceeds. We define scale-up as “the process by which health interventions shown to be efficacious on a small scale and/or under controlled conditions are expanded under real-world conditions to reach a greater proportion of the eligible population, while retaining effectiveness” [3]. Sustained effectiveness and costs associated with scaling-up EBIs are rarely investigated [2, 4]. That oversight needs to be addressed. Thus, researchers are encouraged to identify and justify costs associated with the implementation process and the intervention itself [2].

Among the factors that influence scale-up and sustainment of public health and health-promoting EBIs, funding looms large [5, 6]. Healthcare decision-makers seek to improve health without increasing healthcare budgets. When comparing alternative courses of action, funding partners (often government agencies) increasingly scrutinize investments and consider costs along with other factors such as effectiveness, acceptability in context, scalability, and reach [7]. Health economic evaluation provides a framework to compare the costs and benefits of different alternatives over various time horizons—outcomes of the evaluation inform decision making [8]. Despite its value to decision makers, few studies examined the costs of implementing effective health-promoting EBIs across a long period of scale-up; nor have they studied the economic consequences associated with optimization [4]. We sought to fill this information gap drawing upon our decade of experience implementing and scaling up Choose to Move (CTM).

Choose to Move (detailed below) is a scalable, health-promoting EBI for older adults. CTM was co-designed and scaled up across British Columbia, Canada with community and government partners using a phased approach. Elsewhere, we describe our formal, systematic, and data-driven co-adaptation process prior to implementing CTM across each phase of (horizontal [9]) scale-up [10–12]. We aimed to retain fidelity to the ‘core functions’ of CTM across phases (defined as behavior change techniques that theoretically drive positive health outcomes (e.g., goal setting) [13]). Briefly, we began with a formative evaluation (2015). Phases 1 and 2 comprised the pilot and early scale-up of CTM (2016-17). For Phase 3 (2018-20), CTM was adapted with feedback from older adults and delivery partners to establish “best fit” and to support broad scale-up [10]. In response to COVID-19 public health restrictions (2020), CTM was adapted with delivery partners for delivery in the virtual environment [11]. For Phase 4 (2020-22), CTM was adapted to selectively reduce resource use (program delivery hours by activity coaches); virtual delivery remained an option [12]. We demonstrated that CTM could be implemented with fidelity at increasingly larger scale [14], in different communities, and after adapting for virtual delivery [11] with reduced resource use [12]. Adaptations did not compromise the effectiveness of CTM (i.e., health benefits for older adult participants were retained).

Our current costing study has two objectives: (1) identify, measure, and value the costs of implementing CTM across four phases (7 years) of scale-up; and (2) analyze change in implementation costs alongside changes in intervention effect sizes to assess cost-consequence trends from Phases 1–2 through Phase 4.

## Methods

### Choose to Move

Choose to Move comprises the program (https://choosetomove.ca) and a suite of implementation strategies [15] used to support implementation and scale-up. The CTM program is choice-based, coach- and peer-supported, and designed for low active (< 150 min/week moderate-to-vigorous physical activity (PA)) community-dwelling older adults. Participants are supported by an activity coach to choose physical activities that align with their personal preferences, health status, and available resources [14, 16, 17]. The current 3-month CTM (Phase 4) program comprises a 30 min one-on-one consultation with a trained activity coach, and 8 group meetings with other participants, facilitated by the activity coach. We provide an overview of CTM Phases 1–4 in Supplementary Table 1. Phase 4 continues to be scaled up across British Columbia; to date (August 2025) > 8,400 older adults have participated in CTM.

Implementation [18, 19] and scale-up [20] frameworks and principles (e.g., community partnerships) guide implementation and scale-up of CTM. With government funding, the Active Aging Society (AAS; https://www.activeagingsociety.org) provides broad functional and financial support to community-based delivery partner organizations (e.g., recreation centres, neighbourhood houses) who hire activity coaches to deliver CTM, in person or virtually (e.g., on Zoom™), under the umbrella of their organizations. A Central Support Unit [21], housed within the AAS, uses a suite of implementation strategies to recruit and build capacity (e.g., provides tools, training and ongoing support) among delivery partner organizations. CTM was initially (Phases 1–2) delivered through two large organizations (i.e., YMCA and British Columbia Recreation and Parks Association (BCRPA)) that have broad geographic reach through recreation facilities across British Columbia [14, 16]. Beginning in 2018 (Phase 3+) the AAS recruited small partner organizations (e.g., neighborhood houses and seniors’ centres) whose reach was more local.

### Evaluating CTM

#### Previous studies

Elsewhere, we describe outcomes of the CTM evaluation across all phases of implementation [12, 14, 16, 22]. Briefly, we used a hybrid type 2 effectiveness-implementation study design [23] to evaluate CTM.

For the effectiveness (outcomes) evaluation, participants were > 60 years, able to read and understand English, reported low levels of PA (less than 150 min/week), and had no contraindications to participating in PA. Participants were recruited via local promotions, Facebook, the CTM website, media advertisements and word of mouth. The primary outcome across all phases of CTM was self-reported PA (number of days in the previous week with 30 min or more of moderate-to-vigorous PA) using a validated single-item PA measure [24–26]. Secondary outcomes were mobility, social isolation and loneliness, and health related quality of life. 

Generally, CTM enhanced participants’ engagement in PA, increased mobility and decreased social isolation and loneliness across all phases [12, 14, 16]. We observed a small *voltage drop* [2] in the magnitude of positive change in health outcomes (PA, mobility, loneliness) between Phases 1–2 and Phase 3. However, there was no further ‘scale-up penalty’ [27] for these outcomes between Phases 3 and 4 (except for loneliness in participants aged 75 years and older). In fact, we noted a voltage ‘surge’ in the positive effects of CTM on PA and mobility between Phases 3 and 4 [12]. Outcomes did not differ between CTM delivery modes (in-person or virtual); many health benefits were maintained one year after the end of formal participation in CTM Phases 1–2 and Phase 3 [14, 28].

For the implementation evaluation we were guided by a proposed minimum data set of implementation indicators [29]. Our implementation evaluation methods have changed over time due to changes in CTM program components (e.g., we reduced the number of check-in phone calls after Phase 2 and eliminated them after Phase 3) and our growing interest in other aspects of implementation. This varied measurement approach prevents us from directly comparing implementation across phases. However, in general, implementation was high across all phases: dose delivered (of group meetings) was virtually 100% across all phases, and participant satisfaction with the program was consistently high (> 75%). *Current study*: For the current study, we used data from our two large delivery partners to compare effectiveness across 7 years of CTM scale-up (2016–2022). Specifically, we compared CTM Phase 4 effect sizes (change from baseline with 95% confidence interval, CI) for primary and secondary outcomes to those of Phases 1–2 and Phase 3 effect sizes. We used the approach of McCrabb et al. [27] to determine the proportion of the effect size retained in Phase 4 as compared with Phase 3 as: (Phase 4 effect size/Phases 3 effect size) * 100. We describe percent change narratively. Values greater than 100% indicate a greater benefit of the intervention in Phase 4 as compared with Phase 3; values of 50% indicate that Phase 4 was half as effective as Phase 3; and values less than 0% (negative values) indicate that the direction of the effect in Phase 4 was opposite that of Phase 3. We used the same approach to determine the proportion of effect size retained in Phase 4 as compared with Phases 1–2.

### Cost and cost-consequence analysis

We identified, measured, and valued the costs and outcomes of CTM Phases 1–4, against a ‘do nothing’ alternative. We chose this approach because health promotion activities for older adults are not routinely delivered in Canada. Thus, CTM does not displace usual practice activities or investments in these activities. We adopted a program provider perspective to reflect the real-world implementation of CTM, over a trial time horizon (3 months).

We identified, measured, and valued the economic costs associated with implementation of CTM Phases 1–4. Economic costs include both financial costs (expenses incurred in dollars) and opportunity costs (the value of the forgone opportunity to use those resources for another purpose). In health economic analyses, economic costs are preferred over financial costs for any evaluation intended to inform decisions regarding the optimum allocation of scarce resources and when considering the replicability and sustainability of healthcare interventions [30]. We define implementation costs as all costs associated with delivering CTM and the provision of ongoing implementation support to partner organizations throughout the lifecycle of program delivery (e.g., activity coach hours, equipment and resources, support staff). Financial costs included the costs of goods and services directly purchased to implement CTM which hold a market value. We define opportunity cost as the value of opportunities forgone. That is, any non-financial investment of resources, labor time, or products associated with implementing CTM. We used trial administrative records kept by the Central Support Unit to retrospectively identify resource use associated with implementation of Phases 1–2 across our delivery partners [31]. Delivery partners used a cost-capture tool developed in Microsoft Excel (2013) to prospectively collect cost data for Phase 3 and Phase 4 using a time-driven activity-based costing approach [32]. The cost capture tool included the following categories: (1) labor (staff, including overhead to allow for additional costs of employment), (2) materials such as non-labor cost items (e.g., stationary, education materials, electronic hardware or software), (3) joint costs such as those incurred in connection with multiple projects (e.g., maintenance costs of a website portal supporting different interventions); capital costs such as those for one-off investments (e.g., purchase of computers), and (4) miscellaneous costs (i.e., costs not easily classified into the other categories such as travel, or facilities grants of $500 which were offered to cover costs such as in-house promotion, printing and room booking fees). Our activity-based costing approach involved three parameters: (1) frequency of activity, (e.g., training session, phone call with support staff) (2) resources required to perform one single event of the activity, and (3) price or value of the resources used to perform the activity [32]. Cost data were treated as counts of resource use, weighted by unit costs. The cost for each phase was determined by summing the costs relevant to and coded for that phase. The cost per participant was calculated as the cost of implementing CTM divided by the number of participants. Development, research and evaluation costs were excluded from cost and cost-consequence analyses as we focus on the costs of implementing CTM.

The program provider perspective for this evaluation meant costs to participants and private care providers (including opportunity costs) were not included. Costs incurred after 12-months were not discounted as the trial time horizon was three months and was a function of the study design and not the expected timeline for real world expenses. All costs were adjusted for inflation using annual consumer price index [33] and reported in 2024 Canadian dollars ($CDN).

We conducted a series of trial-based cost and cost-consequence analyses of health outcomes common across CTM Phases 1 through 4. Effectiveness is a prerequisite for economic evaluation; therefore, we included only outcomes that demonstrated a statistically significant effect size [14, 16]. Analyses were performed in R version 4.3.3 using the tidyverse package for data analysis [34]. Results of the cost-consequence analysis are presented as the total cost of CTM alongside the range of outcomes reflected in the primary and secondary trial outcomes (consequences). We conducted the economic evaluation and report findings in accordance with the Consolidated Health Economic Evaluation Reporting Standards (CHEERS) publication guidelines and good reporting practices [8] (Supplementary Table 2).

In practice, there is expected variation around estimates of cost, particularly when programs are implemented in diverse settings, with different program providers, and at scale. To explore the impact of variation on CTM program costs we conducted four scenario analyses to estimate a plausible upper and lower range and demonstrate the impact of uncertainty in key parameters on program delivery costs. Specifically, we calculated the average cost per program and per participant for each program provider (delivery partner). We estimated the range (low and high) of costs by: (1) calculating the range of material costs (years with the least and most number of facilities grants; funds provided to each delivery site (BCRPA sites only) to support program implementation) and (2) calculating the labor costs if Central Support Unit staff time was increased or decreased by 20% (equivalent to 0.2 FTE). We used these estimates to calculate the range of costs per participant and per program in a ‘best case scenario’ (lowest number of facilities grants provided and a 20% reduction in central support staff time) and ‘worst case scenario’ (highest number of facilities grants provided and a 20% increase in central support staff time). We excluded retrospective cost data (Phases 1–2) from the scenario analyses because they provided less detail than did the prospective Phase 3 and Phase 4 data.

## Results

Table 1 describes the number of programs and participants across Phases 1–2, Phase 3 and Phase 4. Table 1 also summarizes the total cost of implementing CTM across all phases and per participant. Per-participant implementation costs were lower in Phase 4, amounting to 37% of the per-participant implementation costs in Phases 1–2. We note a reduction of $803,463 in total costs and $343 in cost per participant between Phase 3 and Phase 4. Variation from differences in delivery partner provided the greatest individual impact on program costs (Range $894 - $1,017). In our scenario analyses, the variation in both facility grants, and staff time had the greatest overall impact on program costs (Range $866 - $1,014).

**Table 1:** The total cost of implementing Choose to Move across all phases and per participant (Canadian 2024 dollars, CDN$), including scenario analyses

**Category**	**Phase 1–2**	**Phase 3**	**Phase 4**	
Participants (*n*)	534	1668	1278	
Programs (*n*)	55	165	135	
	**Phase 1–2**	**Phase 3**	**Phase 4**	**Δ (Phase 1–2 to 4)**
**Total cost (CDN$)**	863,559	1,564,446	760,983	102,576
Average cost per participant	1,617	938	595	1,022
Average cost per program	15,701	9,481	5,637	10,064
	**Phase 3**		**Phase 4**	
**Scenario Analyses**	**Low ($)**	**High ($)**	**Low ($)**	**High ($)**
Delivery Partner				
Average cost per participant	894	1,017	582	626
Average cost per program	8,712	11,020	5,592	5,731
Facility Grants				
Average cost per participant	919	963	587	605
Average cost per program	9,289	9,740	5,554	5,723
Staff Time				
Average cost per participant	908	968	570	621
Average cost per program	9,177	9,786	5,399	5,875
Facilities and Staff Time				
Average cost per participant	866	1,014	562	630
Average cost per program	8,757	10,254	5,316	5,961

Table 2 describes the proportion of CTM implementation costs associated with each resource use category. The evidence-based optimization and reduction in activity coach hours required to deliver Phase 4 resulted in a decrease in total labor costs between Phases 3 and 4 ($992 per program). Cost of materials decreased between Phase 3 and Phase 4, whereas the cost of miscellaneous items increased by 8% ($52,169) as a proportion of the total budget (Table 2). Across all three phases, labor accounted for 63% − 80% of total CTM implementation costs.

**Table 2:** Proportion of Choose to Move implementation costs associated with each resource use (labor, materials, miscellaneous) category. Costs are in Canadian dollars (2024)

	Labor		Materials		Miscellaneous		
	Cost ($)	%	Cost ($)	%	Cost ($)	%	Total cost ($)
Phases 1–2	693,270	80%	119,816	14%	50,473	6%	863,559
Phase 3	981,086	63%	559,614	36%	23,746	2%	1,564,446
Phase 4	565,171	74%	119,897	16%	75,915	10%	760,983

Table 3 describes the comparison of primary outcomes between Phase 3 and Phase 4 and between Phase 1–2 and Phase 4. The effectiveness of CTM Phases 1–2, Phase 3 and Phase 4 for each statistically significant outcome and age group is published elsewhere [12, 14, 16]. We compared effect sizes of CTM Phase 4 outcomes to those of Phases 1–2 and Phase 3 (Table 3). When comparing CTM effect sizes (95% CI) for primary outcomes between Phase 3 and Phase 4, there was no voltage drop in effect size for PA or mobility for the full cohort, or in loneliness for those aged < 75 years. The voltage drop detected in social isolation was 17-38%; for loneliness the voltage drop was 70% for those aged ≥75 years. Between Phases 1–2 and Phase 4, the magnitude of improvement in social isolation improved across the full cohort and both age groups. The magnitude of improvement in PA, mobility, and loneliness decreased across CTM Phases 1–2 to Phase 4, suggesting a voltage drop in effectiveness as CTM was scaled up.

**Table 3:** Comparison of Choose to Move intervention effect sizes (95% CI) for primary and secondary outcomes between Phase 3 and Phase 4 and between Phase 1–2 and Phase 4. Costs are in Canadian dollars (2024)

	Phase 4 Effect Size^a^			Phase 3 to 4: Proportion (%) of CTM effect achieved post-optimization^b^			Phases 1–2 to 4: Proportion (%) of CTM effect achieved with optimization^b^		
	Full cohort	< 75 yrs	≥ 75 yrs	Full cohort	< 75 yrs	≥ 75 yrs	Full cohort	< 75 yrs	≥ 75 yrs
Physical activity	1.3	1.5	1.0	147	150	111	94	94	100
	(1.2, 1.5)	(1.3, 1.6)	(0.7, 1.2)						
Mobility	−0.053	−0.061	−0.034	113	105	131	37	43	24
	(−9.7, −0.01)	(−11.2, −0.01)	(−11.8, 5.1)						
Social isolation	0.58	0.69	0.31	83	86	62	145	115	344
	(0.41, 0.74)	(0.50, 0.89)	(0.002, 0.61)						
Loneliness	−0.15	−0.24	0.02	75	115	30	38	58	6
	(−0.24, −0.07)	(−0.34, −0.13)	(−0.14, 0.19)						

^a^As per McCrabb et al. [28] effect size was calculated as change from baseline. CTM Phase 4 effect sizes are also reported elsewhere [12]

^b^(Phase 4 effect size / Phase 3 effect size) * 100. Values greater than 100% indicate a greater benefit of the intervention in Phase 4 than in Phase 3; values of 50% indicate that Phase 4 was half as effective as Phase 3; and values less than 0% (negative values) indicate that the direction of the effect in Phase 4 was opposite that in Phase 3. The same approach was used to compare Phase 4 with Phases 1-2

## Discussion

As governments and health care decision makers grapple with escalating healthcare costs associated with aging populations, upstream investment in health promotion may provide one solution. To guide decision making, it is imperative that those who fund scaled-up health-promoting EBIs have evidence (data) to support effectiveness, scalability and implementation costs of the intervention. These data are essential to review and prioritize investment decisions. However, very few studies described the cost, cost consequences and effectiveness of health-promoting EBIs as they proceed along the scale-up continuum [35]. Therefore, our study fills this gap. Specifically, we extend our previous work [12] and report costs and cost consequences of an effective health-promoting EBI across 7 years of scale-up.

The CTM context is unique—as we are unaware of any other health-promoting EBIs for older adults that were effectively scaled up with government support over the longer term. This is key, as health-promoting EBIs must be scaled up [36] and sustained [37] to improve health at the population level. Optimizing CTM to reduce cost in Phase 4 while maintaining effectiveness is also a novel aspect of our study.

We also extend the literature and our previous work [12] by identifying, measuring and valuing the financial and opportunity costs associated with implementation of CTM across 7 years of scale-up. Our key finding was that total implementation costs for CTM between Phase 3 and Phase 4 decreased by 37%. Although CTM implementation costs were reduced (due primarily to a 40% reduction in activity coach hours [12], participant-level benefits (PA and mobility in all participants; loneliness in those < 75 years) in Phase 4 were comparable to, or greater than those observed in Phase 3.

Scaled-up, optimized (to reduce cost) health-promoting EBIs should not compromise effectiveness. We reported a small voltage drop between CTM Phases 1–2 and Phase 3 [14]. However, as we continued to scale and optimize CTM in Phase 4 to reduce resource use, effect sizes for physical activity, mobility, and loneliness (in those < 75 years) were similar or greater than those in Phase 3. This finding counters the voltage drop theory, which suggests that as community-based PA [38] and obesity [27] interventions go to scale, the magnitude of their effect diminishes by 50–60%, on average. Despite this, positive changes in most health outcomes remained significant [14]. The difference in these findings across phases may be due, to slightly lower baseline PA in our Phase 4 cohort (2.0 days/week; 95% CI: 1.8, 2.1) who joined CTM during the COVID-19 pandemic, as compared with Phase 3 participants (2.3 days/week; 95% CI: 2.1, 2.6) [12]. The substantial decrease in social isolation (voltage surge) between Phases 1–2 and Phase 3 [14] may reflect how CTM was adapted to create more opportunities for participants to socially connect in group sessions [12, 14]. The voltage drop we observed in social isolation and loneliness between Phase 3 and 4 may be attributed to the public health restrictions in place during the COVID-19 pandemic. We demonstrated that a health-promoting EBI can be optimized for cost while retaining core functions and program effectiveness.

Using economic evidence to inform how best to develop and scale-up an intervention is of great value [39]. Yet little is known about how to evaluate economic aspects of scaling up programs and strategies that comprise health-promoting EBIs [40]. Nor is there much evidence to support how best to use economic data to optimize effective, scalable health-promoting EBIs. Findings from our study in an implementation, health promotion research context, highlight an emergent application of economic analysis. As cost savings were realized for CTM delivery without compromising health benefits, we also urge greater investment in this area of research.

All areas of health and medical research focus on the rigorous measurement of health, program, and (less so) implementation outcomes. However, methods for measuring costs are neither systematic nor comprehensive [2]. Additionally, economic evaluation in public health or health promotion is not routine, and cost data for these interventions are of mixed quality [41]. Poor quality cost data are detrimental, as reliable data form the foundation of economic evaluations. Without reliable estimates, evaluation results could mislead decision makers and have significant implications for healthcare providers and patients [2–4].

Economic evaluation of effectively scaled-up population health EBIs have encountered challenges. First, methods used to evaluate value for money vary [42]. Second, there is little agreement on how best to determine ongoing investment in implementation or scale-up of EBIs [43]. This could be because of different time horizons between conducting public health or health-promoting EBIs and realizing participant-level health benefits. Benefits may also occur outside the healthcare system, which precludes reliably measuring an EBI’s value (or return on investment) to the health system. A third challenge is that differences in healthcare financing and populations assessed limits transferability of cost data to different settings.

Activities that drove successful implementation of CTM were related to building capacity within two larger community organizations that served as delivery partners (i.e., to implement CTM among their older adult constituents). Readiness-building activities [44] were embedded within the essential role of the Central Support Unit. We consider the role of the Central Support Unit as the foundation upon which EBIs are effectively implemented, scaled up, and sustained. This team provided training, tools and ongoing support directly to delivery partners and activity coaches while (indirectly) supporting participants. Thus, it comes as no surprise that as a proportion of the total cost of CTM, labor (including activities of the Central Support Unit) was the largest cost category across all CTM Phases. The Central Support Unit’s role in building capacity, trust, and a sense of community among community partner organizations, activity coaches and participants takes time, often with uncertain outcomes. We have begun to experience that Central Support Unit costs escalate further in smaller more poorly resourced community organizations that are less ‘ready’ [44].

Our study has several strengths. First, we contribute to the scant literature that has determined costs and consequences of health-promoting EBIs across scale-up. We use a rare real-world example of a scaled-up health-promoting EBI (CTM) to derive our data. Second, we specify the cost of resource use within the total cost of implementing CTM. Third, we highlight that CTM can be optimized for cost while not compromising health outcomes; this finding can be applied to other health-promoting EBIs.

We also acknowledge several limitations of our study. First, to minimize the likelihood of missing data in the retrospective costing, all items included in the cost analysis were identified from trial records. Where possible we triangulated data against a second data point or source and confirmed it with the Central Support Unit. Second, we did not impute missing outcomes data. Third, CTM retrospective data (Phases 1–2) did not provide sufficient detail to delineate material costs for research from material costs associated with development. Thus, these data are missing from Table 2 and the scenario analyses.

## Conclusions

Identifying costs associated with scale-up and value for money of health-promoting and public health EBIs is of high priority [45]. We demonstrated an emergent application of economic analysis within scale-up science and contend that investments in advancing this type of research are worthwhile. In order to maximize the public health impact of EBIs while minimizing costs, it is important to understand whether a positive intervention effect (at lower cost) can be sustained over time, and the economic cost of implementation strategies (specifically) used in public health interventions [41]. Although links between scaling up and sustaining EBIs have cost implications, factors and processes that influence both are not well understood. Finally, we urge researchers (including implementation scientists) to identify ways to prospectively weave economic evaluation into implementation, scale-up and sustainment science as part of standard practice. By providing cost and outcomes data, decision makers are better positioned to make informed investments in innovative public health and health-promoting EBIs.

## Supplementary Information

Supplementary Material 1.

## Data Availability

The datasets analyzed during the current study are not publicly available as consent was not obtained for this. However, data are available from the corresponding author on reasonable request.

## Abbreviations

CTM: Choose to Move
CDN$: Canadian dollars
EBI: Evidence-based intervention
PA: Physical activity
AAS: Active Aging Society
YMCA: Young Men’s Christian Association
BCRPA: British Columbia Recreation and Parks Association
CI: Confidence interval
