# Supplementary material for: Are costs optimized as scale-up of Choose to Move–an effective health-promoting intervention for older adults–proceeds?
Source: Int J Behav Nutr Phys Act. 2025 Nov 13;22:144. doi: 10.1186/s12966-025-01826-4 (PMC12613748; doi:10.1186/s12966-025-01826-4)
Supplement: Supplementary file 1 — Supplementary Material 1. [file 12966_2025_1826_MOESM1_ESM.docx]

**Supplementary Table 1.** Key differences between Choose to Move Phases 1-2, Phase 3, and Phase 4. Adapted from Gray et al. (10) and Nettlefold et al. (22) with permission from Springer Nature and Human Kinetics, respectively.

|  | **CTM Phases 1-2** | **CTM Phase 3** | **CTM Phase 4** |
| --- | --- | --- | --- |
| **Activity coach delivery hours** | - 100 hours/program | - 67 hours/program | - 40 hours/program |
| **Program length** | - 6 months | - 6 months | - 3 months |
| **Program components**  **(overview)** | - **Information session:** None - **One-on-one consultation:** 60-min during same week as group meeting 1 - **Group meetings:** 4 (in-person); 60 min - **Check-ins:** 10 (telephone); 15 min, on average; weekly and bi-weekly in months 1-3, monthly in months 4-6 | - **Information session:** 1-2 weeks prior - **One-on-one consultation:** 60-min one week prior to group meeting 1 - **Group meetings:** 5 (in-person); 60 min - **Check-ins**: 6 (telephone, email, in-person); 15 min, on average; once per month | - **Information Session:** 1-2 weeks prior - **One-on-one consultation:** 30-min between group meetings 1 & 2 - **Group meetings:** 8 (in-person and/or virtual); 60 min - **Check-ins:** None. Core functions (e.g., goal setting, action planning, etc.) of the check-ins shifted to the group meetings (specifically, meetings 1, 4, and 8) |
| **Program components (detailed)** | | | |
| **One-on-one consultation** | - - 60-min during same week as group meeting 1   - Activity coach supported goal setting and action planning tailored to participants’ interests, capacities and resources | - - 60-min one week prior to group meeting 1   - Same as Phases 1-2 | - - 30-min between group meetings 1 & 2   - Same as Phases 1-2 |
| **Group meetings** | - - **Group meeting topics** (active travel included in every meeting)     1. Physical activity & chronic conditions     2. Chronic disease self-management     3. Reducing stress & easing anxiety     4. Review CTM principles & behaviour change   - **Movement breaks:** None designated   - **Social interaction:** None designated   - **Group challenges:** None   - **Peer check-ins:** None | - - **Group meeting topics** (active travel in first meeting only)  1. Physical activity & social connection 2. Healthy weight management & nutrition 3. Stress & anxiety 4. Brain health & preventing injury 5. Revisit your goals & celebrate!    - **Movement breaks:** Prescribed for each meeting    - **Social interaction:** Prescribed group and paired discussions; contact information (optional to share)    - **Group challenges:** None    - **Peer check-ins:** None | - - **Group meeting topics** (active travel in group meeting 2 only)  1. Welcome and goal setting 2. Physical activity & social connection 3. Incidental physical activity 4. Goals revisited 5. Nutrition 6. Falls prevention 7. Stress management & brain health 8. Goals and celebration    - **Movement breaks:** Prescribed for in-person meetings; coaches encouraged participants to get up and move around during virtual meetings    - **Social interaction:** Prescribed group and paired discussions; contact information (optional to share)    - **Group challenges:** included at the end of each group meeting    - **Peer check-ins:** Optional |
| **Check-in newsletter (optional)** | - - Not available | - Not available | - Optional (bi-weekly) |
| **Activity coach training** | - - **Qualification: c**ertified fitness leaders or kinesiologists, hired through delivery partner organizations   - **Training**:   - Specific to fitness professionals   - One day, in-person session and a hardcopy manual.   - Social connectedness not formally integrated into the activity coach training | - - **Qualification:** anyone with experience in fitness leadership or with older adults, hired through delivery partners in consultation with recreation coordinators   - **Training**:   - Expanded to support activity coaches who may not be fitness professionals   - Self-directed online platform and interactive practical component   - Enhanced focus on building social connections | - - **Qualification:** anyone with experience in fitness leadership or with older adults, hired through delivery partners in consultation with recreation coordinators   - **Training**:   - Expanded to support activity coaches who may not be fitness professionals   - Self-directed online platform and interactive practical component   - Enhanced focus on building social connections |
| **Program operations** | - - **Lead time:** not standardized, often < 3 months   - **Communication plans:** No formal communication plans   - **Recruitment resource:** No central recruitment resource available to delivery sites   - **Delivery site agreements:** exact roles and responsibilities unclear | - - **Lead time:** every delivery site given 3-6 months lead time   - **Communication plans:** Site-specific communication plan, implementation and site activity checklists   - **Recruitment resource:** Central recruitment resource available to delivery sites ([www.choosetomove.info](http://www.choosetomove.info)); Promotion and recruitment materials modified to highlight the benefits of CTM to participants   - **Delivery site agreements:** modified to more clearly articulate expectations (roles and responsibilities) regarding promotion and recruitment | - - **Lead time:** every delivery site given 3-6 months lead time   - **Communication plans:** Site-specific communication plan, implementation and site activity checklists   - **Recruitment resource:** Central recruitment resource available to delivery sites ([www.choosetomove.info](http://www.choosetomove.info)); Promotion and recruitment materials modified to highlight the benefits of CTM to participants   - **Delivery site agreements:** modified to more clearly articulate expectations (roles and responsibilities) regarding promotion and recruitment |

**Supplementary Table 3**. Consolidated Health Economic Evaluation Reporting Standards (CHEERS) checklist items reported in the economic evaluation of Choose to Move

| **Item** | **Guidance for Reporting** | **Reported in Section** | **Page** |
| --- | --- | --- | --- |
| Title | Identify the study as an economic evaluation and specify the interventions being compared. | 1 | 1 |
| Abstract | Provide a structured summary that highlights context, key methods, results and alternative analyses. | 2 | 2 |
| Background and objectives | Give the context for the study, the study question and its practical relevance for decision making in policy or practice. | 3 | 4, 5 |
| Health economic analysis plan | Indicate whether a health economic analysis plan was developed and where available. | 4 | 5, 6 |
| Study population | Describe characteristics of the study population (such as age range, demographics, socioeconomic, or clinical characteristics). | 5 | 4, 5, 6 |
| Setting and location | Provide relevant contextual information that may influence findings. | 6 | 4 |
| Comparators | Describe the interventions or strategies being compared and why chosen. | 7 | 6 |
| Perspective | State the perspective(s) adopted by the study and why chosen. | 8 | 7 |
| Time horizon | State the time horizon for the study and why appropriate. | 9 | 7 |
| Discount rate | Report the discount rate(s) and reason chosen. | 10 | 7 |
| Selection of outcomes | Describe what outcomes were used as the measure(s) of benefit(s) and harm(s). | 11 | 7, 8 |
| Measurement of outcomes | Describe how outcomes used to capture benefit(s) and harm(s) were measured. | 12 | 7, 8 |
| Valuation of outcomes | Describe the population and methods used to measure and value outcomes. | 13 | 7 |
| Measurement and valuation of resources and costs | Describe how costs were valued. | 14 | 6, 7 |
| Currency, price date, and conversion | Report the dates of the estimated resource quantities and unit costs, plus the currency and year of conversion. | 15 | 7 |
| Rationale and description of model | If modeling is used, describe in detail and why used. Report if the model is publicly available and where it can be accessed. | 16 | NA |
| Analytics and assumptions | Describe any methods for analyzing or statistically transforming data, any extrapolation methods, and approaches for validating any model used. | 17 | 8 |
| Characterizing heterogeneity | Describe any methods used for estimating how the results of the study vary for sub-groups. | 18 | NA |
| Characterizing distributional effects | Describe how impacts are distributed across different individuals or adjustments made to reflect priority populations. | 19 | NA |
| Characterizing uncertainty | Describe methods to characterize any sources of uncertainty in the analysis. | 20 | 8 |
| Approach to engagement with patients and others affected by the study | Describe any approaches to engage patients or service recipients, the general public, communities, or stakeholders (e.g., clinicians or payers) in the design of the study. | 21 | NA |
| Study parameters | Report all analytic inputs (e.g., values, ranges, references) including uncertainty or distributional assumptions. | 22 | 8, 9 |
| Summary of main results | Report the mean values for the main categories of costs and outcomes of interest and summarize them in the most appropriate overall measure. | 23 | 8, 9 |
| Effect of uncertainty | Describe how uncertainty about analytic judgments, inputs, or projections affect findings. Report the effect of choice of discount rate and time horizon, if applicable. | 24 | 10 |
| Effect of engagement with patients and others affected by the study | Report on any difference patient/service recipient, general public, community, or stakeholder involvement made to the approach or findings of the study. | 25 | 11, 12 |
| Study findings, limitations, generalizability, and current knowledge | Report key findings, limitations, ethical or equity considerations not captured, and how these could impact patients, policy, or practice. | 26 | 12 |
| Source of funding | Describe how the study was funded and any role of the funder in the identification, design, conduct, and reporting of the analysis. | 27 | 16 |
| Conflicts of interest | Report authors' conflicts of interest according to journal or International Committee of Medical Journal Editors requirements. | 28 | 16 |
